# AKT Kinase Pathway: A Leading Target in Cancer Research

**DOI:** 10.1155/2013/756134

**Published:** 2013-11-13

**Authors:** Ambuj Kumar, Vidya Rajendran, Rao Sethumadhavan, Rituraj Purohit

**Affiliations:** Bioinformatics Division, School of Bio Sciences and Technology, Vellore Institute of Technology University, Vellore, Tamil Nadu 632014, India

## Abstract

AKT1, a serine/threonine-protein kinase also known as AKT kinase, is involved in the regulation of various signalling downstream pathways including metabolism, cell proliferation, survival, growth, and angiogenesis. The AKT kinases pathway stands among the most important components of cell proliferation mechanism. Several approaches have been implemented to design an efficient drug molecule to target AKT kinases, although the promising results have not been confirmed. In this paper we have documented the detailed molecular insight of AKT kinase protein and proposed a probable doxorubicin based approach in inhibiting miR-21 based cancer cell proliferation. Moreover, the inhibition of miR-21 activation by raising the FOXO3A concentration seems promising in reducing miR-21 mediated cancer activation in cell. Furthermore, the use of next generation sequencing and computational drug design approaches will greatly assist in designing a potent drug molecule against the associated cancer cases.

## 1. Introduction

AKT1, a serine/threonine-protein kinase also known as AKT kinase, is involved in the regulation of various signalling downstream pathways involved in cell metabolism, cell proliferation, survival, growth, and angiogenesis. It is a member of the most frequently activated proliferation and survival pathway in cancer. AKT recognizes and phosphorylates the consensus sequence RXRXX(S/T) of the target proteins when surrounded by hydrophobic residues [1]. The activation of AKT1 is driven by membrane localization, which is in turn initiated by the binding of the pleckstrin homology (PH) domain to phosphatidylinositol-3,4,5-trisphosphate (PtdIns(3,4,5)P3) or phosphatidylinositol-3,4-bisphosphate (PtdIns(3,4)P2), followed by phosphorylation of the regulatory amino acids serine 473 (Ser 473) and threonine 308 (Thr 308) [1]. Because this sequence is present in many proteins, numerous AKT substrates have been identified and validated [2]. Genetic mutations in AKT signalling pathway regulators have been reported to induce oncogenic transformation of the healthy human cell [3] and detected in malignant glioma and endometrial cancer and to some extent in prostate cancer [4, 5], non-small cell lung cancer [6], melanoma [7], hepatocellular carcinoma [8], and breast cancer [9]. The importance of AKT in human cancer is largely inferred from frequently occurring mutations in the enzymes that regulate the activity of these second messenger phospholipids (PtdIns(3,4,5)P3, PtdIns(3,4)P2) and ultimately cause the activation of AKT through membrane recruitment [1]. Tumour samples from the patients with breast, colorectal cancer and cases of leukaemia have been shown to frequently harbour activating somatic mutations in AKT1 [1]. Germline mutations in the AKT pathway regulators have been detected with high rate in the autosomal dominant hamartoma cancer syndromes [10], Cowden disease [11], Bannayan-Zonana syndrome [12], and Lhermitte-Duclos disease [13]. Hence, AKT1 seems to have a crucial but passive role in oncogenesis and acts as an indirect intermediary between mutated upstream regulatory proteins and downstream signalling molecules [1].

The oncogenic activation of AKT1 can be induced by several means, most commonly occurring either due to the compromise in its membrane targeting by PH domain or due to the pathological conformational changes occurring in the mutant structure [1]. The genetic mutations in PH domain have been previously reported to interfere with correct localization and sensitivity towards the PtdIns and have led to major consequences to its functional behaviour [1]. A point mutation at nucleotide 49 that results in a lysine substitution for glutamic acid at amino acid 17 (AKT(E17 K)) has been implicated in cancer cases [1]. While screening the causes behind such observation, the computational approach forms a significant backbone and serves in carrying keen experimental observations in low cost input. Thus, in one of our works, we conducted molecular docking and molecular dynamics simulation to infer the associated molecular changes occurring in AKT1 PH domain [1]. The results showed that the mutation induces rapid conformational drifting in the PH domain which might be the crucial reason behind the loss of AKT1/2 inhibitor VIII interaction and of a 4-fold rise in its localization to plasma membrane [1].

**Figure 1:**
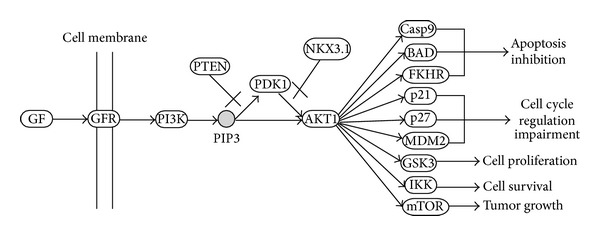
Regulation of AKT signalling activation.

Regulation of AKT signalling activation is carried out by the transfer of phosphate group from PI(3,4,5)P3 to AKT protein (Figure 1). PI(3,4,5)P3 is a lipid second messenger involved in phosphate mediated activation of various downstream effectors associated with the oncogenic pathways [14]. Their role in regulation of AKT/PKB kinase and in amplifying AKT induced antiapoptotic and growth stimulatory effects has been supported by several research articles [15]. AKT is the important downstream target of PI(3,4,5)P3, which controls cell proliferation and protects the cell from apoptosis. The negative regulation of phosphate mediated AKT signalling pathway through phosphatase activity of PTEN [16] and NKX 3.1 [17] helps in maintaining a feedback control towards the phosphate transfer and associated activation of oncogenic pathways. The loss of their phosphatase activity prevents the dephosphorylation of phosphatidylinositol-3,4,5-trisphosphate to phosphatidylinositol-4,5-bisphosphate, which then allows the transfer of phosphate molecule to PDK1 and AKT proteins, allowing the activation of MDM2, GSK3, P27, P21, CASP9, BAD, FKHR, IKK, and MTOR genes (Figure 1). This activation finally leads to the apoptosis inhibition [15], cell cycle progression [18], tumour growth [19], and impairment of G1 and G2 cycle arrest [20].

## 2. miRNA Based Gene Expression Regulation

Other than genetic mutations, role of miRNAs have also been identified as the active mediator of tumorigenic cellular transformations, targeting the 3′-UTR region of the tumour suppressor genes [21]. MicroRNAs that are partially complementary to a target can also speed up deadenylation, causing mRNAs to be degraded sooner. miRNAs occasionally also cause histone modification and DNA methylation of promoter sites [22, 23], which affects the expression of target genes. PTEN, NKX 3.1, and PTENP1 are the well-known AKT signalling pathway regulators and are also the favourite site for miRNA's regulated deactivation [24]. PTEN and NKX 3.1 are the known targets of multiple miRNAs including, most notably, the glioma-implicated miR-21 [25]. Furthermore, miR-26a has also been identified as an active candidate in downregulating the PTEN expression in breast and prostate cancers [26]. hsa-miR-22, another mature miRNA, is actively involved in forming a regulatory loop in PTEN/AKT pathway and modulates signalling kinetics, downregulating the PTEN expression levels by acting directly through a specific site on PTEN 3′-UTR [27]. Moreover, hsa-miR-1297, hsa-miR-19, hsa-miR-22, and hsa-miR-23ab are also involved in oncogenic downregulating of PTEN expression in human cells [28, 29]. The in-depth understanding of these miRNAs and their role in suppressing the gene activity can help to inhibit the phosphate mediated oncogenic AKT signalling pathway by targeting the AKT and PI3 K genes using specific miRNAs, thus protecting cell from the rapid tumorigenic proliferations. Moreover, it can become the future endeavour in finding a promising cure to the associated cancer cases. These approaches in coordination with other target based drug therapies can prove to be an asset to future cancer research.

## 3. Genomic Variations

Genomic variations, especially in the exonic regions have been identified as the key factor in inducing cancers in human. Through the advancements of genome sequencing technologies, we have now become highly capable of identifying these oncogenic mutations, and it has paved our way to understand their possible role in inducing cancers. Illumina HiSEQ and Solexa 3D machines along with the excellent data analysis computational platforms have now enabled us to conduct high range genome wide association studies (GWAS) and to develop the target based drug therapies. Potential implementation of genome sequencing technology in studying the gene downregulation and in studying the exact mechanism of their downregulation by elucidating its causal element has been a great achievement in the field of cancer research [30]. Research carried out by Astle et al. (2012) using NGS technology has presented promising results in understanding the role of NGS in identifying the target site in oncogenic AKT signalling pathway for drug discovery [31]. Further, the gene expression analysis using mRNA sequencing has provided an additional support to understand the effectiveness of genome sequencing as an effective tool in cancer researches [32]. The role of NGS technology is becoming the center of excellence in cancer research day by day, facilitating the candidate gene identification in various forms of cancers [33]. It also provides the possible way to design target based therapies by identifying the intercellular components to target the tumour promoting genes [34, 35]. Detecting the role of hsa-miR-7 in controlling the tumour progression in non-small cell lung cancer using NGS techniques has provided us an excellent insight in developing the microRNA based therapy for cancer control [36].

## 4. Current Approach

In an effort to improve therapeutic options in cancer, many investigational drugs are being developed to inhibit signalling pathways that promote the survival of cancer cells [37, 38]. To date, the most developed inhibitor of AKT is perifosine, a lipid-based inhibitor. In vitro, perifosine inhibits translocation of AKT to the cell membrane and inhibits the growth of melanoma, lung, prostate, colon, and breast cancer cells [38–40]. Synergistic effects of perifosine and traditional chemotherapeutic agents such as etoposide in leukemia cells [41], doxorubicin in MM cells [42], and temozolomide in glioma cells [43] is also a notable element that can be used to design a potent drug molecule. Moreover, perifosine has also been found to sensitize cancer cells to apoptosis and cell cycle arrest induced by radiation in vitro and in vivo [44–46]. Recently, it was shown that perifosine leads to significant inhibition of proliferation and induction of apoptosis in Waldenstrom macroglobulinemia cells in vitro [47]. Furthermore, triciribine (API-2), also known as triciribine-phosphate, was identified as an AKT inhibitor after screening the National Cancer Institute (NCI) structural diversity set. Triciribine inhibits AKT2 phosporylation at both sites (T309 and S474) and inhibits EGF-induced phosphorylation of all three isoforms of AKT in vitro [48]. More recently, rapamycin analogues such as CCI-779 and RAD-001 have been explicitly designed for development as anticancer drugs [49, 50]. These inhibitors of mTOR bind to the FK506-binding protein, FKBP-12, which then binds and inhibits mTOR [49, 50]. Synergistic effects of rapamycin and EGFR TKIs have been observed in several in vitro systems, including glioblastoma multiforme [51–53], prostate cancer [54, 55], pancreatic cancer [54], squamous cell carcinoma [56], renal cell carcinoma [57, 58], leukemia [59], cervical carcinoma [60], and non-small cell lung cancer [54]. Several other studies extended the efficacy of these combinations to xenograft experiments [54, 60, 61]. Some of the most commonly used Akt inhibitors are listed in Santa Cruz Biotechnology (http://www.scbt.com/chemicals-table-akt_inhibitors.html). Although combinations of these pathway inhibitors with various types of chemotherapy have been conducted extensively in preclinical studies, only a few of them have been able to minimize the tumour growth and provide a permanent cure to cancer, where the patient selection and toxicity test prevail as a major hurdle.

## 5. Computational Approaches for Drug Design

The protein 3D structure forms a major drug targeting element in pharmacological studies, and most of the drug discovery methods rely on the structural conformations of target proteins. Conformational flexibility of a protein molecule affects its interaction with a ligand and their biological partners at different levels [62–73]. At a particular time step a particular protein attains specific conformation that occupies a minimum on its free-energy landscape. Transitions from one minimum to another correspond to dynamic changes in the structure of the protein that controls their continuous structural fluctuations and is central to protein function. In silico approaches provide an excellent platform to determine these conformation properties of proteins. Advancements in computing power, systematic tools, and algorithms have improved the quality of protein structure simulation and analysis to a very high extent. In silico molecular modelling when combined with molecular dynamics simulation approaches helps in identifying the stable conformation and significant structures that can be used to study the consequences of structural variants.

Molecular dynamics simulation (MDS) is one of the principal tools in the theoretical study of biological molecules. This computational method calculates the time dependent behaviour of a molecular system. MD simulations have aided in gaining the detailed insight of the atomic fluctuations and conformational changes of proteins and nucleic acids. These methods are now routinely used to investigate the structure, dynamics, and thermodynamics of biological molecules and their complexes. The MDS techniques are also very useful in detecting the changes in protein conformation and atomic fluctuations. Molecular dynamics simulation approaches have also been extensively used to report the structural consequences of the cancer associated point mutations. The native and mutant structures are imposed to the long-term molecular dynamics simulation in order to record the changes in their motion trajectory. Atomic fluctuations, structural changes, domain loss, changes in the vital protein folds, and stability, as well as the retention and loss of crucial interactions, can be easily studied using the MDS approach. The root mean square deviation (RMSD), root mean square (RMSF), radius of gyration (Rg), solvent accessible surface area (SASA), principal component analysis (PCA), energy change, dihedral changes, and DSSP calculations are some of the most crucial factors that have enabled us to determine the in-depth structural consequences of the cancer associated mutations. Moreover, the in silico docking experiments are usually followed by MDS of the protein-drug complex molecule. This helps in detecting the stability of the protein-drug complex which further helps in determining the effectiveness of drug in binding to a particular target protein. Certain high range force fields that have provided a wide range of options to simulate a protein structure in different environment have now become the central criterion for structure analysis and drug design.

## 6. Suggested Approach

Through the advancement in the use of genome sequencing techniques in clinical bioinformatics and target based drug therapy development, the accuracy in the postclinical trials has raised in the last few decades. The use of such techniques to unravel the mechanism behind miR-21 mediated PTEN gene silencing and to find a plausible cure can be a better approach. Moreover, doxorubicin, a potential drug which translocates the FOXO3A protein inside the cell nucleus [74], in combination with rapamycin, can prove to be a potential combination to diminish the proliferation of miR-21 induced cancer cell proliferation and could be a better template to study the oncogenic miR-21 pathway inactivation. FOXO3A attacks the promoter binding region of the miR-21 coding region in TEMM43 gene, which in turn deactivates the proliferation of this miRNA. As discussed above, the high level of miR-21 diminishes the activity of PTEN by binding it at 3′-UTR region which plays an active role in inducing cancer proliferations. Inhibiting the miR-21 activation by raising the FOXO3A concentration could help in reducing miR-21 mediated cancer activation in cell. Many other targets in AKT kinase pathway are available which can be exploited to study the cancer cell proliferation mechanism and can be further used to find the cure.
